# mDIXON-Quant technique diagnostic accuracy for assessing bone mineral density in male adult population

**DOI:** 10.1186/s12891-023-06225-z

**Published:** 2023-02-14

**Authors:** Rui Tang, Guangyu Tang, Ting Hua, Yun Tu, Rui Ji, Jingqi Zhu

**Affiliations:** Department of Radiology, Shanghai Tenth People’s Hospital, Tongji University School of Medicine, 301 Middle Yanchang Road, Shanghai, 200072 China

**Keywords:** Chemical shift encoded, Quantitative computed tomography, Dual-energy X-ray absorptiometry, Fat fraction map, T2^*^ map, Bone mineral density, Male adults

## Abstract

**Background:**

To investigate the diagnostic efficacy of mDIXON-Quant technique for prediction of bone loss in male adults.

**Methods:**

One hundred thirty-eight male adults were divided into normal, osteopenia, and osteoporosis groups based on DXA and QCT for the lumbar spine. Differences in mDIXON-Quant parameters [fat fraction (FF) and T2^*^ value] among three groups, as well as the correlation of mDIXON-Quant parameters and bone mineral density (BMD) were analyzed. The areas under the curves (AUCs) for mDIXON-Quant parameters for prediction of low bone mass were calculated.

**Results:**

According to DXA standard, FF and T2^*^ value were significantly increased in osteoporosis group compared with normal group (*P* = 0.012 and* P* < 0.001). According to QCT standard, FF was significantly increased in osteopenia and osteoporosis groups compared with normal group (both *P* < 0.001). T2^*^ values were significantly different among three groups (all *P* < 0.05). After correction for age and body mass index, FF was negatively correlated with areal BMD and volumetric BMD (*r* = -0.205 and -0.604, respectively; both *P* < 0.05), and so was T2^*^ value (*r* = -0.324 and -0.444, respectively; both *P* < 0.05). The AUCs for predicting low bone mass according to DXA and QCT standards were 0.642 and 0.898 for FF, 0.648 and 0.740 for T2^*^ value, and 0.677 and 0.920 for both combined, respectively.

**Conclusions:**

FF combined with T2^*^ value has a better diagnostic efficacy than FF or T2^*^ value alone in prediction of low bone mass in male adults, which is expected to be a promising MRI method for the screening of bone quality.

**Trial registration:**

ChiCTR1900024511 (Registered 13–07-2019).

## Background

Osteoporosis (OP) is a systemic metabolic disease characterized by decreased bone mass and destruction of bone microarchitecture with high risk of fragility fractures [1]. With the increasing aging, OP has become one of the major diseases affecting the health of middle-aged and elderly population. Therefore, accurate prediction of low bone mass is particularly important to prevent the occurrence of fragility fractures at an early stage.

Bone densitometry is the primary method for diagnosing OP, predicting fragility fractures, and a reliable basis for monitoring the efficacy of treatment. The methods for measuring bone mineral density (BMD) are mainly dual-energy X-ray absorptiometry (DXA) and quantitative computed tomography (QCT). DXA, as a traditional measurement method, has some advantages including less radiation, low cost, convenience, and the ability to measure multiple parts. However, DXA is susceptible to a variety of factors such as obesity, spinal degenerative disease and aortic calcification, which may lead to false negative or false positive. Compared to DXA, QCT reduces the influence of the above factors. QCT is considered to be a more accurate technique to reflect real change of bone mass. However, the increased radiation dose limits the clinical application of QCT for screening OP. Besides, many studies have pointed out that a single measurement of bone mass is not enough to assess the bone strength in recent years [2, 3].

Iron overload was shown to result in decreased bone formation, suggesting that iron can negatively affect osteoblast function, which may ultimately lead to iron-related osteoporosis [4]. Recent studies have shown that an increase of marrow adipose tissue (MAT) can also reduce bone mass [5–7], which suggests that MAT and iron deposition can be used as early indicators for monitoring changes in bone marrow microenvironment. Previous studies mainly used proton magnetic resonance spectroscopy (^1^H-MRS) technique to quantify the MAT [8]. However, unlike mature applications in non-bone marrow tissues or organs, the instability of results limited the application of ^1^H-MRS technique in bone marrow. T2^*^ magnetic resonance imaging (MRI) has been widely used to quantify iron deposition in the liver and myocardium. The detection of iron by T2^*^ MRI is based on the paramagnetic effect produced by the deposited iron [9, 10]. With the development of functional MRI, chemical shift encoded (CSE) was gradually used to quantify the fat content and iron deposition in the whole body including bone marrow. Based on CSE technique, modified Dixon quantification (mDIXON-Quant) sequence designed by Philips Healthcare has been widely used for the diagnosis of diseases such as fatty liver, with the advantages of short scanning time, simple post-processing, and accurate quantification of fat and iron deposition [11, 12]. Previous MAT studies have demonstrated a strong correlation between mDIXON-Quant and ^1^H-MRS [13, 14]. Some studies have used DXA or QCT techniques to obtain BMD to reveal the characteristics of mDIXON-Quant parameters in middle-aged and elderly populations (especially postmenopausal women) with varying bone mass [15]. However, studies investigating the relationship between CSE parameters and BMD (both measured by DXA and QCT) in male adults are rare [16]. Therefore, the application value of CSE technique is not clear in male adult population.

High-end MRI scanners are common in general hospitals in Shanghai, China. The cost of MRI examination is not a burden for patients due to the payment of government medical insurance. These factors create the conditions for the implementation of mDIXON-Quant technique, especially for the elder patients who have a pre-existing need for routine lumbar MRI. In addition to predicting bone mass, this technique also reflects bone marrow microenvironment, which can not be assessed by DXA or QCT.

The aim of this study is to investigate the diagnostic efficacy of mDIXON-Quant technique for prediction of BMD based on DXA and QCT standards in male adult population.

## Materials and methods

### Subjects

One hundred fifty six subjects were consecutive Chinese male adults who visited Shanghai Tenth People’s Hospital for annual physical examination (welfare of enterprise) from April 2018 to July 2021. The inclusion criteria were as follows: (1) patient aged ≥ 18 years; (2) voluntary participation in DXA, QCT and MRI examinations of the lumbar spine. The exclusion criteria were as follows: (1) metabolism disorders including hyperparathyroidism, diabetes, Cushing's syndrome, and renal osteodystrophy; (2) use of drugs that affect bone metabolism including corticosteroids, calcitonin, calcium, vitamin D, diphosphonates, and estrogen; (3) compression fracture, bone marrow edema and inflammation, relatively large Modic changes, tumor-like lesions and benign bone tumors, and malignancies of lumbar spine which may affect measurement; (4) history of malignancy, regardless of whether the patient had received treatment including surgery, radiotherapy, chemotherapy, and immunotherapy; (5) bedridden for more than 1 week during the last 3 months; (6) patient received spinal surgery; (7) history of lumbar trauma during the last 3 months; (8) patient had contraindications of DXA, QCT or MRI examinations; and (9) images with poor quality which may affected observation and measurement. A total of 138 subjects were finally included in the study. This prospective study was approved by the ethics committee of Shanghai Tenth People’s Hospital (Number: SHSY-IEC-4.1/18–200/01) and registered on the Chinese Clinical Trials Registry (Number: ChiCTR1900024511). Written informed consent was obtained from all patients.

### Imaging technique

#### DXA examination

The areal BMD [aBMD, defined as grams per square centimeter (g/cm^2^) of calcium hydroxyapatite] of cancellous bone of vertebral body was measured from lumbar 1 (L1) to L4 by DXA (QDR4500, HOLOGIC, USA). The mean aBMD value of four vertebrae (L1-L4) was taken and the T score was recorded. The scanning parameters were as follows: voltage 76 kV, current 3.0 mA, scan length 20.2 cm, scan width 18.0 cm. Subjects were divided into normal group [T score ≥ -1.0 standard deviation (SD)], osteopenia group (-2.5 SD < T score < -1.0 SD) and OP group (T score ≤ -2.5 SD) according to the diagnostic criteria of DXA [17].

### QCT examination

The volumetric BMD [vBMD, defined as milligrams per cubic centimeter (mg/cm^2^) of calcium hydroxyapatite] of cancellous bone of vertebra body was measured from L1 to L3 by QCT (Somatom Force, Siemens Healthcare, Forchheim, Germany) with a solid CT calibration phantom (Mindways software Inc., Austin, TX, USA) placed under the lumbar spine. The scanning parameters were as follows: tube voltages 120 kV, tube current 125 mAs, slice thickness 5 mm, reconstructed slice thickness 1.5 mm. The images of the lumbar spine were transferred to a workstation (Mindways QCT Pro 5.10, Austin, TX, USA) for analysis. ROIs were drawn encompassing the largest region of the cancellous bone of vertebral bodies (L1-L3) on the midplanes of transversal, sagittal, and coronal images to calculate the average vBMD. The measurement of ROI should not be affected by the cortical bone, the basal vertebral vein and other benign lesions. Based on the average vBMD, subjects were divided into normal group (≥ 120 mg/cm^3^), osteopenia group (80–120 mg/cm^3^) and OP group (≤ 80 mg/cm^3^) according to ACR criteria [18].

### MRI examination

MRI examination of the lumbar spine was performed using a 3 T MR scanner (Ingenia, Phillips, Amsterdam, Netherlands) with a body coil. The time interval from DXA/QCT to MRI examination is no more than 2 h. The detailed scanning parameters of conventional and mDIXON-Quant sequences for lumbar spine were summarized in Table 1. The fat fraction (FF) and T2^*^ value of the cancellous bone of the L1-5 vertebrae were measured on FF and T2^*^ maps to quantify fat and iron deposition accordingly at the workstation (ISP V7). The size, shape, and location of the ROIs on the sagittal images of FF and T2^*^ maps were consistent with those on the sagittal image of QCT. The mean FF and T2^*^ values of L1-3, L1-4, and L1-5 were taken for each subject to perform MRI-QCT comparison, MRI-DXA comparison, and repeatability assessment accordingly.

**Table 1:** Magnetic resonance imaging scanning sequence parameter

Parameter	T1WI	T2WI	T2WI-SPAIR	mDIXON-Quant
Scan plane	Sagittal	Sagittal	Sagittal	Sagittal
Time to repetition (ms)	400	2000	2500	5.6
Time to echo (ms)	9	90	80	0.95,1.65,2.35, 3.05,3.75,4.45
No.slices	11	11	11	95
Slice thicknes (mm)	4	4	4	4
Interslice gap (mm)	0.4	0.4	0.4	-2
Field of view (mm^2^)	160 × 280	160 × 280	160 × 280	400 × 350
Acquisition matrix	180 × 276	200 × 320	200 × 309	156 × 135
Voxel size (mm^3^)	0.9 × 1.0 × 4.0	0.8 × 0.8 × 4.0	0.8 × 0.9 × 4.0	2.6 × 2.6 × 4.0
Phase encoding direction	H > > F	H > > F	H > > F	A > > P
NSA	1	1	1	1
Flip angle (degrees)	80	90	90	3
Bandwidth (hertz/pixel)	335	335	335	2428
Acquisition time (min:s)	1:47	2:06	1:45	0:18

*A* Anterior, *F* Feet, *H* Head, *mDIXON-Quant* Modified Dixon quantification, *NSA* Number of signals averaged, *P* Posterior, *SPAIR* Spectral attenuated inversion recovery, *T1WI* T1 Weighted imaging, *T2WI* T2 Weighted imaging

### Repeatability assessment

Thirty patients were randomly selected from the subjects and measured by two radiologists (Zhu J and Tang G, who had more than 15 years of experience in musculoskeletal radiology) using the same method. The radiologists did not have access to clinical information (DXA and QCT) that could bias results. After 7-day interval, Zhu J repeated the measurement for the same patients. Intraclass correlation coefficient (ICC) test was used to calculate inter- and intra-observer agreement. All the data would have been measured independently by Zhu J if a good agreement had been found.

### Statistical analysis

Sample size was predefined by statistical software (PASS 2021, v21.0.5, NCSS Company, USA). The analyses were performed by statistical software (SPSS 25.0, SPSS, Chicago, III). Receiver-operating characteristic (ROC) analysis was performed by Medcalc version 15.6 (MedCalc Software, Mariakerke, Belgium). The normality analysis of continuous data was performed by the Shapiro–Wilk test. The descriptive statistics of normal variables were expressed as mean ± standard deviation. The differences among three groups in terms of normal variables were compared through One-way ANOVA. The descriptive statistics of nonparametric variables were expressed as median (interquartile range). ICC assessed inter- and intra-observer variability of measurements between two radiologists. The differences among three groups in terms of nonparametric variables were compared through Kruskal–Wallis H test. The Pearson’s and Spearman’s correlation coefficient were applied for parametric and nonparametric distribution variables, respectively. The concordance between DXA and QCT for the diagnosis of low bone mass (including osteopenia and OP) was tested using Kappa value without adjustment (≤ 0.4 being poor concordance, 0.4–0.75 being fair concordance, and ≥ 0.75 being good concordance). ROC analysis was performed to evaluate the diagnostic efficacy of FF and T2^*^ value for differentiating between normal bone mass and low bone mass as well as osteopenia and OP. *P* value less than 0.05 was considered statistically significant.

## Results

### Repeatability analysis

ICC test showed that inter- and intra-observer variability of measurements between two radiologists for the DXA, QCT, and mDIXON-Quant parameters presented good agreements varying from 0.942 to 0.988 and from 0.944 to 0.998, accordingly (Table 2).

**Table 2:** Repeatability of radiological measurements

ICC/Parameter	aBMD (g/cm^2^)	vBMD (mg/cm^3^)	FF (%)	T2^*^ value (ms)
Inter-observer	0.980	0.988	0.953	0.942
Intra-observer	0.998	0.988	0.955	0.944

*aBMD* Areal bone mineral density, *FF* Fat fraction, *ICC* Intraclass correlation coefficient, *vBMD* Volumetric bone mineral density

### Clinical and radiological characteristics of participants

**Fig. 1:**
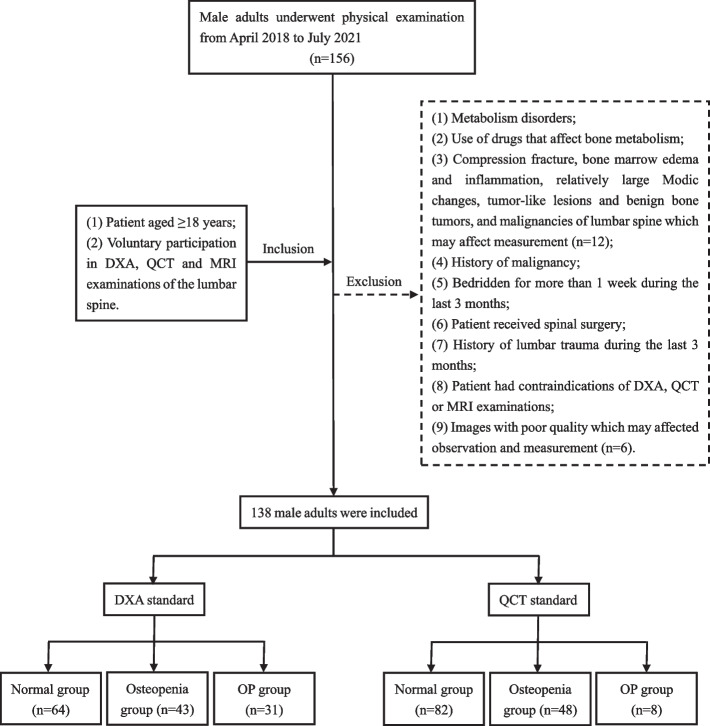
Flow chart of patient enrolment

Flowchart and demographic features of participants are presented in Fig. 1 and Table 3 accordingly. The alternative diagnosis to misleading results in mDIXON-Quant examination included modic changes (especially type 2) and hemangioma which could be differentiated by conventional MRI and QCT. There were no adverse events / drawbacks with mDIXON-Quant examination in this study.

**Table 3:** Clinical and radiological characteristics of all subjects

**Parameter**	**All subjects**	**DXA as reference standard**				**QCT as reference standard**			
		**Normal** **(*n*=64)**	**Osteopenia** **(*n*=43)**	**Osteoporosis** **(*n*=31)**	***P*-Value**	**Normal** **(*n*=82)**	**Osteopenia** **(*n*=48)**	**Osteoporosis** **(*n*=8)**	***P*-Value**
Age (y)	57.0(46.8,62.3)	54.0(44.0,63.0)	54.0(47.0,59.0)	59.0(56.0,65.0)	0.029	51.0(40.8,59.0)	58.0(54.3,65.0)	66.0(59.3,67.8)	＜0.001
BMI (kg/m^2^)	23.9(22.2,26.3)	24.7(23.2,26.9)	23.2(21.3,24.6)	23.5(20.9,24.8)	0.001	24.3(22.4,27.1)	23.7(22.1,24.7)	24.4(21.1,25.2)	0.198
T-score	-1.2±1.4	-0.1(-0.7,0.6)	-1.6(-2.0,-1.3)	-2.8(-3.2,-2.6)	＜0.001	-0.8(-1.4,0.1)	-2.1(-2.8,-1.2)	-2.5(-2.9,-2.4)	＜0.001
aBMD (g/cm^2^)	0.96±0.15	1.08(1.01,1.15)	0.91(0.86,0.95)	0.78(0.74,0.81)	＜0.001	1.01(0.94,1.10)	0.86(0.79,0.96)	0.82(0.78,0.84)	＜0.001
vBMD (mg/cm^3^)	134.1±36.6	157.0(129.0,181.4)	127.6(99.7,153.5)	102.2(85.4,113.8)	＜0.001	157.7（139.3，177.1）	103.0（95.2，113.5）	69.8（56.8，79.2）	＜0.001
FF (%)	51.3±9.3	48.4±9.7	51.8±7.7	54.1±8.9	0.011	47.2(40.2,51.3)	55.7(52.4,61.1)	61.7(51.6,66.2)	＜0.001
T2^*^ value (ms)	7.1(6.1,9.5)	6.6(5.5,7.8)	7.1(6.0,8.9)	9.1(6.6,12.1)	0.001	6.1(5.4,7.2)	7.9(6.3,9.2)	13.0(9.5,18.8)	＜0.001

Data are expressed as mean ± standard deviation or median (interquartile range).

*aBMD A*real bone mineral density, *BMI* Body mass index, *DXA* Dual-energy X-ray absorptiometry, *FF* Fat fraction, *QCT* Quantitative computed tomography, *vBMD* Volumetric bone mineral density

### Diagnostic concordance between DXA and QCT for low bone mass

The Kappa value for diagnostic concordance between DXA and QCT for low bone mass (T score < -1.0 SD for DXA or vBMD < 120 mg/cm^3^ for QCT) was 0.428.

### Comparison of mDIXON-Quant parameters among different age groups

All subjects were divided into young adult group (18 years ≤ age < 40 years; n = 20), middle-aged group (40 years ≤ age < 60 years; *n *= 79), and elderly group (age ≥ 60 years; *n* = 39).

FF was significantly increased in middle-aged and elderly groups compared with young adult group (both *P* < 0.001), and not between middle-aged group and elderly group (*P* = 0.271). T2^*^ value was also significantly increased in middle-aged and elderly groups compared with young adult group (*P* = 0.041 and 0.001), and not between middle-aged group and elderly group (*P* = 0.167) (Table 4).

**Table 4:** Comparison of mDIXON-Quant parameters among varying age

Parameter/Group	Young adult *n* = 20	Middle-aged *n* = 79	Elderly *n* = 39
Age (y)	30.5(27.3,34.8)	54.0(49.0,58.0)	65.0(63.0,68.0)
P-value^*^	< 0.001^a^	< 0.001^b^	< 0.001^c^
FF (%)	42.1(36.2,47.6)	52.0(48.5,55.8)	54.9(49.8,63.1)
P-value^#^	< 0.001^a^	0.271^b^	< 0.001^c^
T2^*^ value (ms)	6.4(5.0,6.9)	7.0(6.1,9.3)	8.5(6.7,10.7)
P-value^&^	0.041^a^	0.167^b^	0.001^c^

Data are expressed as mean ± standard deviation or median (interquartile range)

*FF* fat fraction, *mDIXON-Quant* modified Dixon quantification

^a^ Young adult group vs. middle-aged group

^b^ Middle-aged group vs. elderly group

^c^ Young adult group vs. elderly group

^*^ P-value for age in comparison between different groups

^#^ P-value for FF value in comparison between different groups

^&^ P-value for T2^*^ value in comparison between different groups

### Correlation between mDIXON-Quant parameters and age

FF and T2^*^ values were both poorly correlated with age (*r* = 0.411 and 0.371, both *P* < 0.001).

### Comparison of mDIXON-Quant parameters among different bone mass groups

**Fig. 2:**
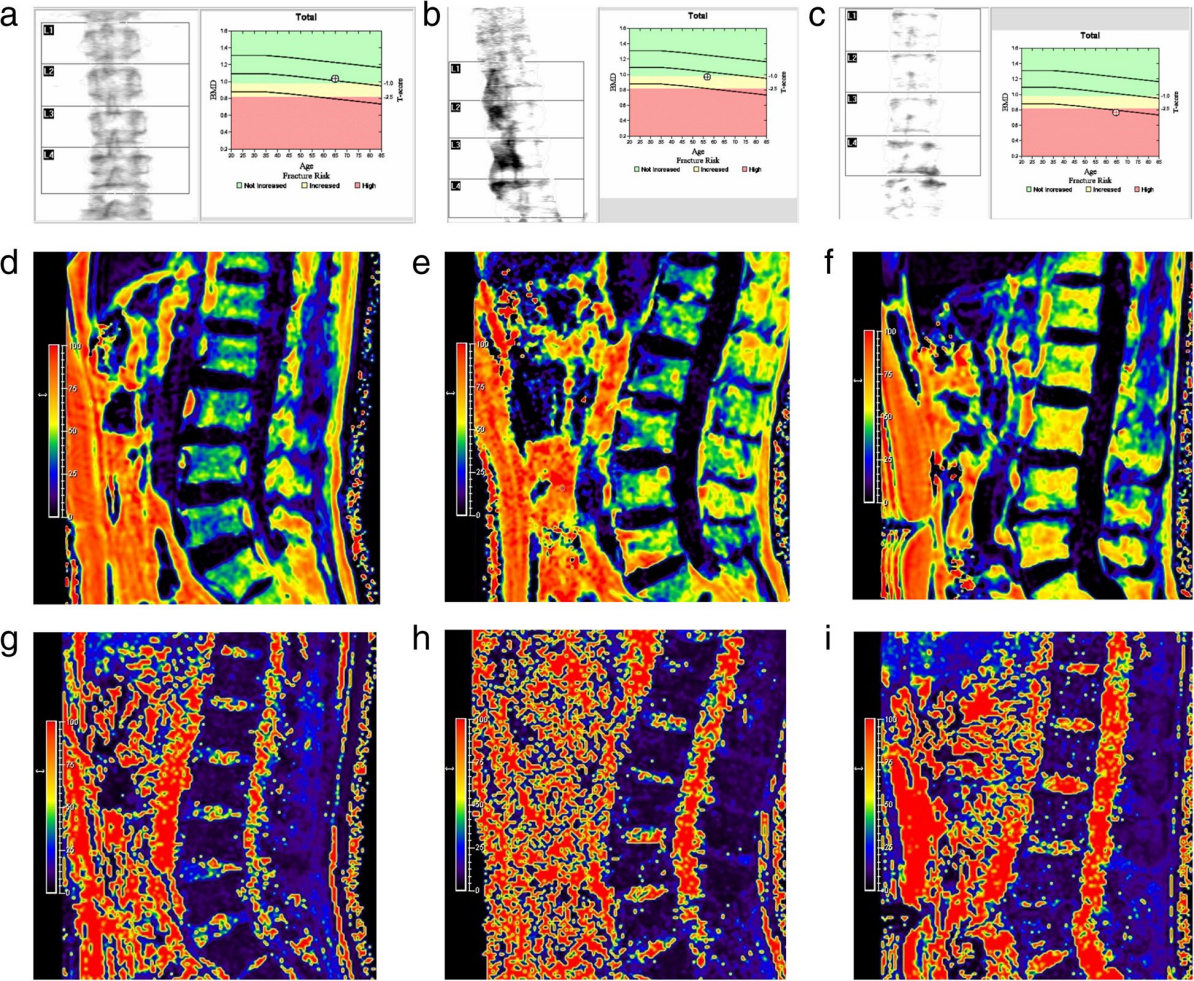
DXA imagings (**a-c**), FF maps (**d-f**) and the corresponding T2^*^ maps (**g-i**) of three male adults with varying bone mass according to the DXA standard. As the color of the lumbar vertebral body changes from purple to red on the FF and T2^*^ maps, it indicates a corresponding increase in marrow adipose tissue and iron. (**a**,** d**, and **g**) A 65-year-old male with nomal bone mass (T score = -0.5). The FF and T2^*^ values were 38.6% and 6.8 ms. (**b**,** e**, and **h**) A 58-year-old male with osteopenia (T score = -1.1). The FF and T2^*^ values were 45.3% and 7.6 ms. (**c**,** f** and **i**) A 65-year-old male with osteoporosis (T score = -2.9). The FF and T2^*^ values were 57.3% and 13.4 ms

According to the DXA standard, FF and T2^*^ values were significantly increased in OP group compared with normal group (*P* = 0.012 and* P* < 0.001), while no significant difference was found in FF and T2^*^ values between normal group and osteopenia group (both *P* > 0.05), as well as between osteopenia group and OP group (both *P* > 0.05) (Table 5; Fig. 2).

**Table 5:** Comparison of mDIXON-Quant parameters among varying BMD

**Parameter/Group**	**aBMD**			**vBMD**		
	**Normal** ***n* = 64**	**Osteopenia** ***n* = 43**	**Osteoporosis** ***n* = 31**	**Normal** ***n* = 82**	**Osteopenia** ***n* = 48**	**Osteoporosis** ***n* = 8**
FF (%)	48.4 ± 9.7	51.8 ± 7.7	54.1 ± 8.9	47.2(40.2,51.3)	55.7(52.4,61.1)	61.7(51.6,66.2)
P-value^#^	0.170^a^	0.808^b^	0.012^c^	< 0.001^a^	1.000^b^	< 0.001^c^
T2^*^ value (ms)	6.6(5.5,7.8)	7.1(6.0,8.9)	9.1(6.6,12.1)	6.1(5.4,7.2)	7.9(6.3,9.2)	13.0(9.5,18.8)
P-value^&^	0.456^a^	0.061^b^	< 0.001^c^	0.001^a^	0.020^b^	< 0.001^c^

Data are expressed as mean ± standard deviation or median (interquartile range)

*aBMD* Areal bone mineral density, *BMD* Bone mineral density, *FF* Fat fraction, *vBMD* Volumetric bone mineral density

^a^ Normal group vs. osteopenia group

^b^ Osteopenia group vs. osteoporosis group

^c^ Normal group vs. osteoporosis group

^#^ *P*-value for FF value in comparison between different groups

^&^ *P*-value for T2^*^ value in comparison between different groups

**Fig. 3:**
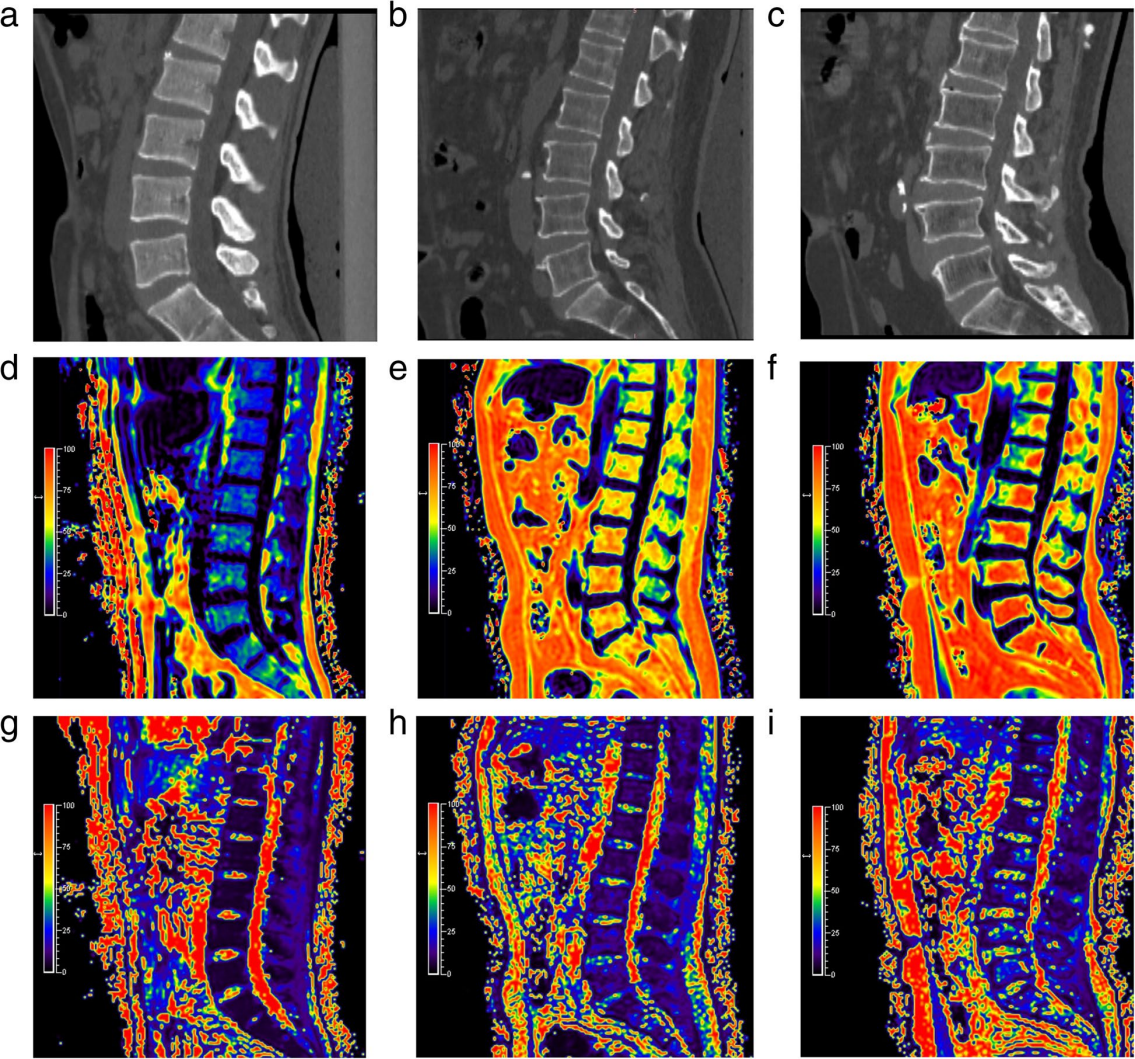
QCT imagings (**a-c**), FF maps (**d-f**) and the corresponding T2^*^ maps (**g-i**) of three male adults with varying bone mass according to the QCT standard. As the color of the lumbar vertebral body changes from purple to red on the FF and T2^*^ maps, it indicates a corresponding increase in marrow adipose tissue and iron. (**a**,** d**, and **g**) A 27-year-old male with nomal bone mass (vBMD = 174.5 mg/cm^3^). The FF and T2^*^ values were 30.8% and 4.8 ms. (**b**,** e**, and **h**) A 65-year-old male with osteopenia (vBMD = 99.1 mg/cm^3^). The FF and T2^*^ values were 65.5% and 9.9 ms. (**c**,** f** and **i**) A 68-year-old male with osteoporosis (vBMD = 41.2 mg/cm^3^). The FF and T2^*^ values were 74.5% and 18.7 ms

According to the QCT standard, FF was significantly increased in osteopenia and OP groups compared with normal group (both *P* < 0.001), and not between osteopenia group and OP group (*P* = 1.000). T2^*^ values were significantly different between any two of the three groups (normal vs. osteopenia,* P* = 0.001; normal vs. OP, *P* < 0.001; and osteopenia vs. OP,* P* = 0.020) (Table 5, Fig. 3).

### FF and T2* values were inversely correlated to both aBMD and vBMD

**Fig. 4:**
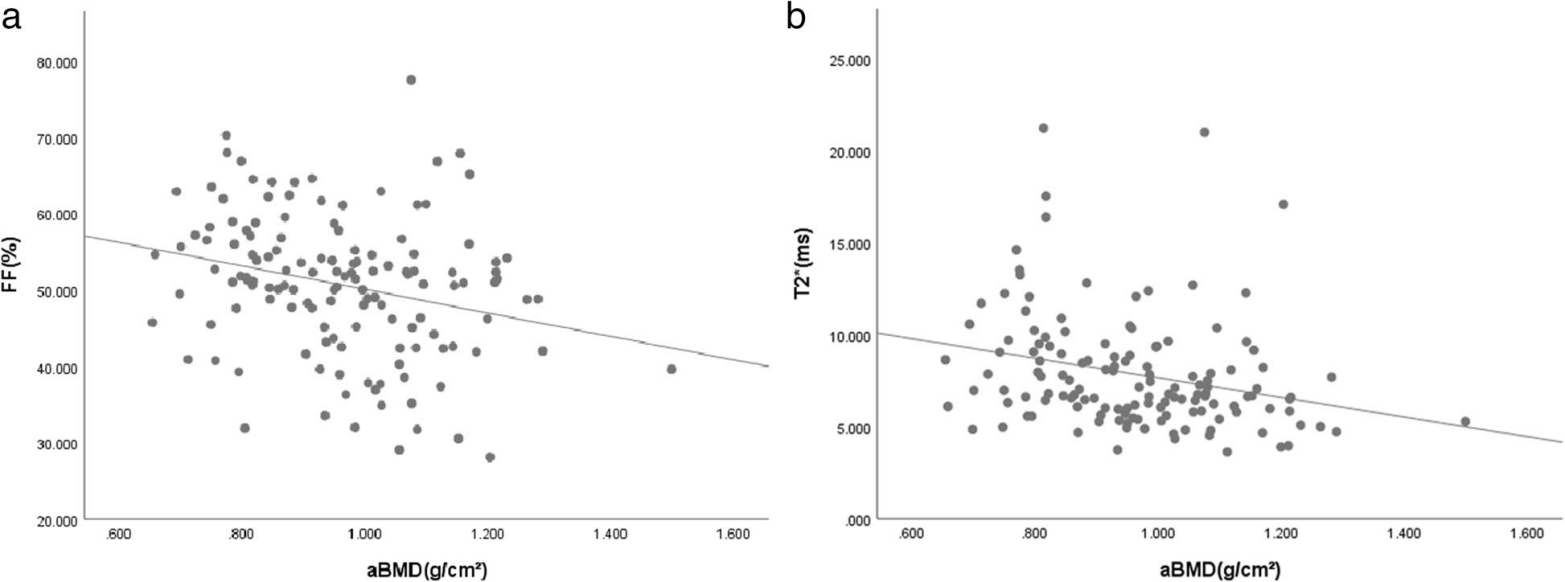
Correlation between mDIXON-Quant parameters and aBMD. The less aBMD, the more FF (**a**; *r* = -0.258) and T2* values (**b**; *r* = -0.324)

FF was inversely correlated with aBMD [*r* = -0.258, *P* = 0.002; *r* = -0.205, *P* = 0.016 after correction for age and body mass index (BMI)]. T2^*^ value was also inversely correlated with aBMD (*r* = -0.324, *P* < 0.001; *r* = -0.324, *P* < 0.001 after correction for age and BMI) (Fig. 4).

**Fig. 5:**
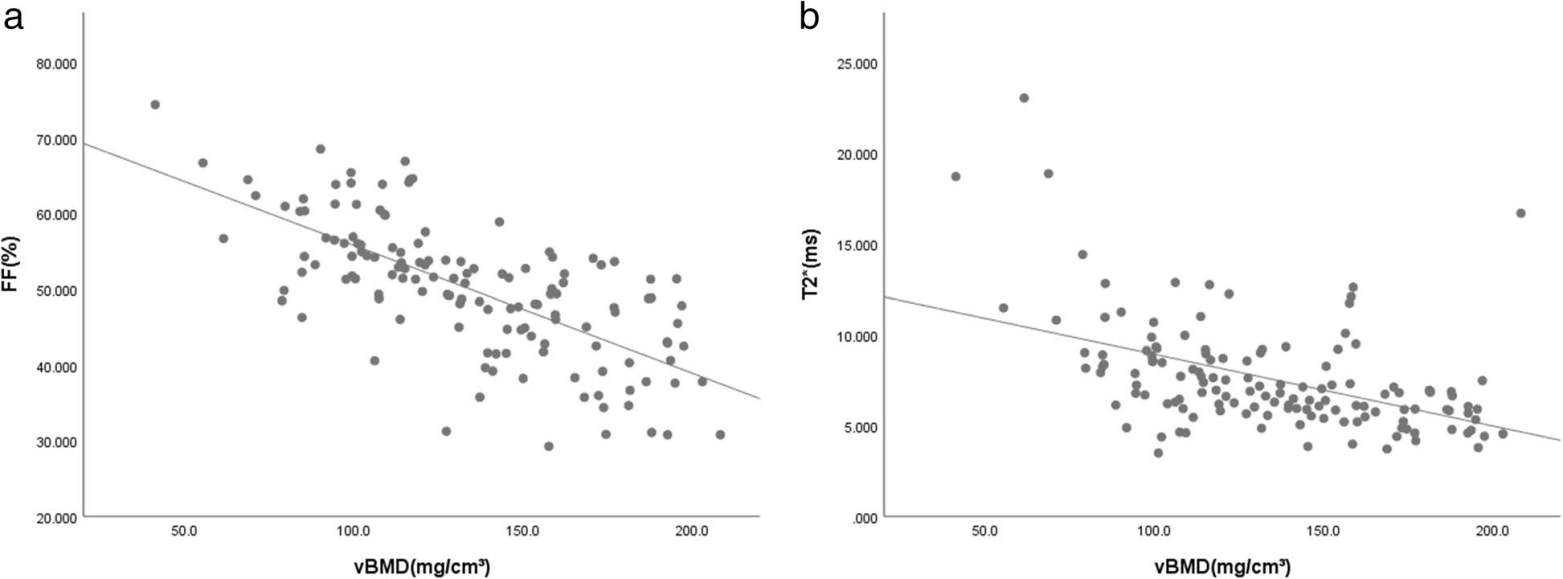
Correlation between mDIXON-Quant parameters and vBMD. The less vBMD, the more FF (**a**; *r* = -0.693) and T2* values (**b**; *r* = -0.506)

FF was inversely correlated with vBMD (*r* = -0.693, *P* < 0.001; *r* = -0.604, *P* < 0.001 after correction for age and BMI). T2^*^ value was also inversely correlated with vBMD (*r* = -0.506, *P* < 0.001; *r* = -0.444, *P* < 0.001 after correction for age and BMI) (Fig. 5).

### Correlation between FF and T2^*^ value in different BMD groups

In normal-osteopenia and OP groups based on DXA standard, FF values were both poorly correlated with T2^*^ values (*r* = 0.330, *P* = 0.001;* r* = 0.391, *P* = 0.030). After correction for age and/or BMI, the results of correlation analyses on FF and T2* values are presented in Table 6**.**

**Table 6:** Correlation between FF and T2^*^ value in varying BMD

**Parameter**	**aBMD**		**vBMD**	
	**Normal-osteopenia**	**Osteoporosis**	**Normal-osteopenia**	**Osteoporosis**
r-value	0.330	0.391	0.246	0.190
P-value	0.001	0.030	0.005	0.651
r-value (adjustment for age)	0.191	0.280	0.072	0.220
P-value (adjustment for age)	0.050	0.135	0.414	0.635
r-value (adjustment for BMI)	0.312	0.355	0.182	0.303
P-value (adjustment for BMI)	0.001	0.054	0.039	0.508
r-value (adjustment for age and BMI)	0.233	0.283	0.098	0.294
P-value (adjustment for age and BMI)	0.017	0.137	0.272	0.572

*aBMD* Areal bone mineral density, *BMD* Bone mineral density, *FF* Fat fraction, *vBMD* Volumetric bone mineral density

In normal-osteopenia group based on QCT standard, FF was poorly correlated with T2^*^ value (*r* = 0.246, *P* = 0.005). However, FF exhibited no significant correlation with T2* value in OP group based on QCT standard (*r* = 0.190, *P* = 0.651). After correction for age and/or BMI, the results of correlation analyses on FF and T2* values are presented in Table 6**.**

### Diagnostic efficacy analysis of mDIXON-Quant parameters

**Fig. 6:**
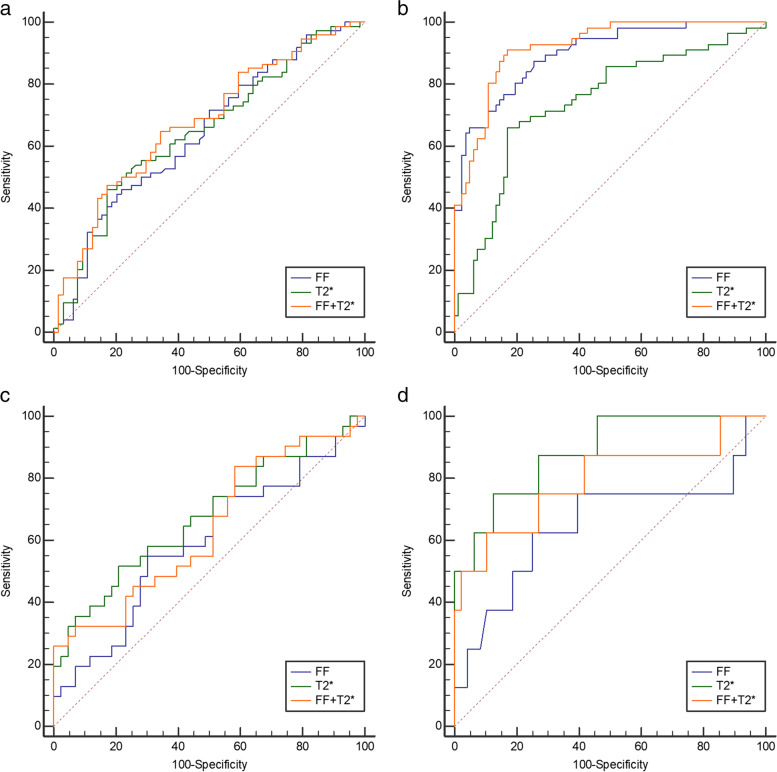
Receiver-operating characteristic curves of mDIXON-Quant parameters for discrimination between normal and low bone mass (**a** and **b**) as well as between osteopenia and osteoporosis (**c** and **d**) according to the DXA (**a** and **c**) and QCT (**b** and **d**) standards

The areas under the curves (AUCs) to differentiate between normal bone mass and low bone mass according to the DXA standard were 0.642, 0.648, and 0.677 for FF, T2^*^ value, and both combined accordingly (Table 7 Fig. 6a).

**Table 7:** Receiver-operating characteristic curve parameters of FF and T2^*^ values for discrimination between normal and low bone mass

Parameter	DXA as reference standard			QCT as reference standard		
	FF	T2^*^ value	FF + T2^*^ value	FF	T2^*^ value	FF + T2^*^ value
AUC (95% CI)	0.642(0.556–0.722)	0.648(0.562–0.728)	0.677(0.592–0.754)	0.898(0.835–0.943)	0.740(0.658–0.811)	0.920(0.861–0.959)
P-value	0.0025	0.0016	< 0.001	< 0.001	< 0.001	< 0.001
Cutoff	53.9%	8.3 ms	0.53191	50.9%	7.6 ms	0.36448
Sensitivity (%)	44.59	45.95	64.86	87.5	66.07	91.07
Specificity (%)	79.69	82.81	65.62	74.39	82.93	82.93
PPV (%)	71.7	75.6	68.6	70.0	72.5	78.5
NPV (%)	55.4	57.0	61.8	89.7	78.2	93.2

*AUC* Area under the curve, *CI* Confidence interval, *DXA* Dual-energy X-ray absorptiometry, *FF* Fat fraction, *NPV* Negative predictive value, *PPV* Positive predictive value, *QCT* Quantitative computed tomography

The AUCs to differentiate between normal bone mass and low bone mass according to the QCT standard were 0.898, 0.740, and 0.920 for FF, T2^*^ value, and both combined accordingly (Table 7 Fig. 6b).

The AUCs to differentiate between osteopenia and OP according to the DXA standard were 0.593, 0.675, and 0.637 for FF, T2^*^ value, and both combined, accordingly (Table 8 Fig. 6c).

**Table 8:** Receiver-operating characteristic curve parameters of FF and T2* values for discrimination between osteopenia and osteoporosis

Parameter	DXA as reference standard			QCT as reference standard		
	FF	T2^*^ value	FF + T2^*^ value	FF	T2^*^ value	FF + T2^*^ value
AUC (95% CI)	0.593(0.472–0.705)	0.675(0.556–0.780)	0.637(0.517–0.746)	0.650(0.511–0.772)	0.885(0.772–0.955)	0.792(0.662–0.889)
P-value	0.1754	0.0069	0.0391	0.2385	< 0.001	0.0048
Cutoff	54.38%	8.978 ms	0.757	60.48%	10.7 ms	0.98675
Sensitivity (%)	54.84	51.61	25.81	62.50	75.00	62.50
Specificity (%)	69.77	79.07	100.00	75.00	87.50	89.58
PPV (%)	56.7	64.0	100.0	29.4	50.0	50.0
NPV (%)	68.2	69.4	65.2	92.3	95.5	93.5

*AUC* area under the curve, *CI* confidence interval, *DXA* dual-energy X-ray absorptiometry, *FF* Fat fraction, *NPV* Negative predictive value, *PPV* Positive predictive value, *QCT* Quantitative computed tomography

The AUCs to differentiate between osteopenia and OP according to the QCT standard were 0.650, 0.885, and 0.792 for FF, T2^*^ value, and both combined, accordingly (Table 8 Fig. 6d).

The cutoff, sensitivity, specificity, positive predictive value, and negative predictive value of FF and T2^*^ value for discrimination between normal and low bone mass as well as between osteopenia and OP according to the DXA and QCT standards are presented in Tables 7 and 8.

## Discussion

The human bone marrow accounts for about 85% of the bone cavity, including the red bone marrow and the yellow bone marrow, which is dominated by hematopoietic cells and adipocytes, accordingly. The changes of BMD and MAT in postmenopausal women are more obvious than men due to a significant decrease in estrogen. MAT was higher in men than in women until the age of 55 years, increased sharply between 55 and 65 years in women, whereas it increased slowly throughout life in men [19].With increasing age, the less bone mass elder population has, the more fat and iron within bone marrow appears [15, 20, 21].

Our study found a borderline agreement between DXA and QCT in diagnosing low bone mass. The positive rate of QCT was lower than that of DXA (40.6% vs. 53.6%) in detecting low bone mass. The accuracy of DXA may be compromised by a variety of factors such as obesity, spinal degenerative disease, aortic calcification, and vertebral compression fractures. Li et al. [22] reported that QCT had a significantly higher detection rate of OP than DXA in postmenopausal women. They found that some patients with a negative diagnosis by DXA and a positive diagnosis by QCT had developed vertebral compression fractures. Yu et al. [23] did an simulation experiment to investigate the effect of increased body fat on DXA and QCT measurements, which indicated that the fat layer significantly reduced lumbar BMD measured by DXA while slightly increasing lumbar BMD measured by QCT. Since the patients of our study were mainly middle-aged and elderly men (85.5%) who were prone to suffer from abdominal obesity, which was the likely cause of measurement difference in low bone mass between DXA and QCT.

BMD is the main determinant of bone strength, reflecting 75–90% of bone strength [24]. However, the assessment of bone mass using BMD alone does not fully reflect bone strength. CSE, a water–lipid separation technique, is a low flip angle, six-echo, seven-peak fat profile method including T2^*^ and novel eddy current compensation that delivers accurate and reproducible quantification of fat and iron deposition in the bone marrow in a single breathhold [25]. CSE techniques are now available on the majority of clinical MRI systems with a different name (mDIXON-Quant, Philips; Q-Dixon, Siemens; and IDEAL, GE), which were widely used in bone quality assessment [15, 26–28].

In our study, FF and T2^*^ values gradually increased with age (especially middle-aged and elderly men) and showed a positive correlation with age, which could be attributed to the accumulation of MAT and iron with increasing age [15, 20]. However, the correlation coefficients between mDixon-Quant parameters and age were lower than those of the reported studies [15, 20]. The differences in characteristics of enrolled patients and study protocols may be the main reason for the discrepancy.

In recent years, some studies have been performed to quantitatively assess FF and T2^*^ values for lumbar OP. Kühn et al. [29] reported that FF was significantly increased in patients with OP and R2^*^ value (R2^*^ value = 1/T2^*^ value) was significantly decreased in patients with osteopenia or OP compared with healthy population, who employed DXA as reference standard in the elderly population (45.1% male). Also, significant differences were found in FF and T2^*^ values between the three QCT-BMD groups in the postmenopausal women [28, 30]. Our results were virtually consistent with the above studies [28–30]. The extensive data on the use of CSE MRI indicated that FF and T2^*^ values were promising biomarker for OP and bad bone quality. However, the differences in the comparison results of these two parameters among three BMD groups based DXA and QCT were observed in our study. The technical advantage of QCT that make it superior to DXA in reflecting bone mass and quality is the key reason for this phenomenon. It is worth noting that both DXA and QCT have quite similar average or median for FF and T2* values to detect normal BMD based on the results of this study, which supports DXA's ability to be used as a screening tool.

Previous studies found a negative correlation between FF acquired from MAT ^1^H-MRS and BMD [13, 31]. Some MAT studies using CSE technique reported that FF and T2^*^ values were inversely correlated with BMD [15, 26, 27]. Our male-only study showed that a gradual increase in FF and T2^*^ values with decreasing BMD obtained from DXA and QCT with or without correction for age and BMI, which was consistent with the previous studies [24, 32, 33]. Our study showed a weak negative correlation between FF and aBMD (*r* = -0.258), which was lower than the result (*r* = -0.459) of a postmenopausal female study [27]. However, relatively high and negative correlation coefficients after adjustment were found,between FF and vBMD (*r* = -0.604) and between T2^*^ value and vBMD (*r* = -0.444) in this study, which was slightly lower than the reported result (*r* = -0.747 between FF and vBMD and *r* = -0.498 between T2^*^ value and vBMD) from 105 postmenopausal female subjects [28]. The discrepancy may be due to differences in study population and protocol. Although many studies have reported that both FF and T2* values increase with decreased bone mass [28–30], the correlation between these two parameters in the bone marrow are rare presented. İdilman et al. [34] reported that no significant correlation was observed between vertebral bone marrow R2^*^ and FF values. In our study, no significant correlation or poor correlation coefficients were found between FF and T2* value in different BMD groups. Multicenter studies to confirm the correlation between the two parameters in the bone marrow is needed.

In a postmenopausal female study conducted by Li et al. [28], the AUC values of FF, T2^*^ vaule, and FF + T2^*^ vaule for predicting low bone mass measured by QCT were 0.894 (cutoff value = 54.65%), 0.852 (cutoff value = 8.25 ms), and 0.944, accordingly. However, lower discriminating ability of CSE parameters (0.748, 0.589, and 0.758 for the AUC values of FF, T2^*^ vaule and FF + T2^*^ vaule; 55.10% and 10.35 ms for the cutoff values of FF and T2^*^ vaule) between osteopenia and OP subjects was observed in the above study. Our male study presented similar results which implies that combined two-parameter model could give better results in discriminating normal and low bone mass by both DXA and QCT. Due to the small number of OP subjects in this study, the true ability of CSE parameters to discriminate between osteopenia and OP needs to be further confirmed by large sample size.

There are several limitations in our study. Firstly, the sample size was relatively small, especially in patients with OP, which resulted in the inability of this study to analyze osteopenia and OP separately. Secondly, the bone marrow adiposity and iron deposition not only varies with age and sex but also varies with respect to the region of interest in the skeleton. Our study emphasizes the findings for the average lumbar spine FF and T2^*^ value only. Spatial distribution differences in FF and T2^*^ value among varying spine levels are not studied. Thirdly, this study lacks validation to demonstrate the clinical value of the mDixon-Quant parameters in predicting low bone mass in male adults. Therefore, large sample size and multicenter study including validation is required in the future.

In conclusion, the mDixon-Quant technique can simultaneously quantify FF and T2^*^ value of the lumbar vertebrae to reflect changes in MAT and iron deposition. FF combined with T2^*^ value has a better diagnostic efficacy than FF or T2^*^ value alone in prediction of low bone mass in male adults, which is expected to be a promising MRI method for the screening of bone quality.

## Data Availability

The datasets generated and/or analyzed during the current study are not publicly available due to patients’ confidentiality but a coded copy of the dataset is available to all public upon request to the corresponding author.

## Abbreviations

aBMD: Areal bone mineral density
AUC: Areas under the curve
BMD: Bone mineral density
BMI: Body mass index
CSE: Chemical shift encoded
DXA: Dual-energy X-ray absorptiometry
FF: Fat fraction
^1^H-MRS: Proton magnetic resonance spectroscopy
ICC: Intraclass correlation coefficient
MAT: Marrow adipose tissue
mDIXON-Quant: Modified Dixon quantification
MRI: Magnetic resonance imaging
OP: Osteoporosis
QCT: Quantitative computed tomography
ROC: Receiver-operating characteristic
vBMD: Volumetric bone mineral density
